# Adherence to Antiretroviral Therapy (ART) in Yaoundé-Cameroon: Association with Opportunistic Infections, Depression, ART Regimen and Side Effects

**DOI:** 10.1371/journal.pone.0170893

**Published:** 2017-01-31

**Authors:** Julius Y. Fonsah, Alfred K. Njamnshi, Charles Kouanfack, Fang Qiu, Dora M. Njamnshi, Claude T. Tagny, Emilienne Nchindap, Léopoldine Kenmogne, Dora Mbanya, Robert Heaton, Georgette D. Kanmogne

**Affiliations:** 1 Department of Neurology, Yaoundé Central Hospital, Yaoundé, Cameroon; 2 Faculty of Medicine and Biomedical Sciences, University of Yaoundé I, Yaoundé, Cameroon; 3 HIV-Day Care Service, Yaoundé Central Hospital, Yaoundé, Cameroon; 4 Department of Biostatistics, College of Public Health, University of Nebraska Medical Center, Omaha, NE, United States of America; 5 Yaoundé University Teaching Hospital, Yaoundé, Cameroon; 6 Department of Psychiatry, University of California San Diego, 9500 Gilman Drive, La Jolla, CA, United States of America; 7 Department of Pharmacology and Experimental Neuroscience, College of Medicine, University of Nebraska Medical Center, Omaha, NE, United States of America; University of Missouri Kansas City, UNITED STATES

## Abstract

Following global efforts to increase antiretroviral therapy (ART) access in Sub-Saharan Africa, ART coverage among HIV-infected Cameroonians increased from 0% in 2003 to 22% in 2014. However, the success of current HIV treatment programs depends not only on access to ART, but also on retention in care and good treatment adherence. This is necessary to achieve viral suppression, prevent virologic failure, and reduce viral transmission and HIV/AIDS-related deaths. Previous studies in Cameroon showed poor adherence, treatment interruption, and loss to follow-up among HIV+ subjects on ART, but the factors that influence ART adherence are not well known. In the current cross-sectional study, patient/self-reported questionnaires and pharmacy medication refill data were used to quantify ART adherence and determine the factors associated with increased risk of non-adherence among HIV-infected Cameroonians. We demonstrated that drug side-effects, low CD4 cell counts and higher viral loads are associated with increased risk of non-adherence, and compared to females, males were more likely to forego ART because of side effects (p<0.05). Univariate logistic regression analysis demonstrated that subjects with opportunistic infections (on antibiotics) had 2.42-times higher odds of having been non-adherent (p<0.001). Multivariable analysis controlling for ART regimen, age, gender, and education showed that subjects with opportunistic infections had 3.1-times higher odds of having been non-adherent (p<0.0003), with significantly longer periods of non-adherence, compared to subjects without opportunistic infections (p = 0.02). We further showed that compared to younger subjects (≤40 years), older subjects (>40 years) were less likely to be non-adherent (p<0.01) and had shorter non-adherent periods (p<0.0001). The presence of depression symptoms correlated with non-adherence to ART during antibiotic treatment (r = 0.53, p = 0.04), and was associated with lower CD4 cell counts (p = 0.04) and longer non-adherent periods (p = 0.04). Change in ART regimen was significantly associated with increased likelihood of non-adherence and increased duration of the non-adherence period. Addressing these underlying risk factors could improve ART adherence, retention in care and treatment outcomes for HIV/AIDS patients in Cameroon.

## Introduction

Of the 37 million individuals worldwide living with HIV/AIDS, 25.8 million (70%) are in Sub-Saharan Africa (SSA) [1]. Since the year 2000, over 25 million people have died from HIV/AIDS, mostly in SSA [1]. With this high HIV/AIDS-related mortality, there has been global efforts to make ART more affordable and increase treatment access to more HIV-infected subjects [2]. Thanks to those efforts many more HIV-infected people in low and middle-income countries are now receiving ART. One such example is Cameroon where the number of HIV-infected subjects receiving ART increased from 28,000 in 1998 to about 200,000 in 2013 [3], the percentage of pregnant women receiving ART increased from 14% in 2009 to 61% in 2013 [4], and overall ART coverage among Cameroonians living with HIV/AIDS increased from 0% in 2003 to 22% in 2014 [5, 6].

The new World Health Organization (WHO) HIV treatment guidelines have expanded the number of people eligible for ART [7] and this expansion is expected to increase ART coverage in several countries, including Cameroon. In fact, through the “Treatment 2015” program, WHO and UNAIDS intend to mobilize support to accelerate and scale-up HIV treatment worldwide, prioritizing countries where 9 out of every 10 people have unmet ART needs [8]. Twenty of those priority countries are in SSA and include Cameroon [8].

Achieving the WHO 2020 treatment goals, and the goal of ending the AIDS epidemic as a public health threat by 2030 [7], will depend on the success of current HIV treatment programs. Such success will not only depend on access to HIV treatment, but also on good adherence to ART and retention in care, which is necessary to achieve viral suppression, to prevent viral failure, diminish viral transmission, and reduce HIV/AIDS-related deaths.

Previous studies of HIV+ subjects on ART in Cameroon showed high rates of non-adherence, treatment interruption, and loss to follow-up [9–14], and this was associated with drug resistance and virologic failure [10, 15]. However, the factors that hinder adherence to ART in Cameroon are not well known. For ART treatment programs in Cameroon to be successful, it is critical to identify barriers to adherence, then determine and implement appropriate measures to promote and improve adherence. In the current study, we used both patient self-reported questionnaires and pharmacy medication refill data to quantify adherence to ART, and to determine the factors associated with increased risk of non-adherence.

## Materials and Methods

### 2.1. Study Design and Ethical Consideration

This cross-sectional study was part of an ongoing project aimed at analyzing the influence of HIV genetic diversity on viral neuropathogenesis in Cameroon. This study was performed in accordance with guidelines of the Helsinki Declaration and was approved by the Cameroon National Ethics Committee, as well as the Institutional Review Board of the University of Nebraska Medical Center. Written informed consent was obtained from all the participants and data were processed using unique identifiers to ensure confidentiality.

### 2.2. Participants

A total of 171 HIV-infected individuals were consecutively recruited between 2008 and 2015 from: 1) the HIV voluntary counseling and testing sections of the Day-care Service in the Yaoundé Central Hospital; 2) the Yaoundé Jamot Hospital; 3) the Efoulan District Hospital, Yaoundé; and 4) the Etoug-Ebe Baptist Health Center, Yaoundé. The purpose of the study and research procedures were fully explained to participants and adults at least 18 years old who gave a written consent were allowed to participate in the study. The exclusion criteria were: 1) present or past history of CNS disease unrelated to HIV, 2) head trauma, 3) current alcohol intoxication (blood alcohol content of each participant was measured using a Breathalyzer), 4) known psychiatric disease or treatment with antipsychotic drugs, and 5) ongoing systemic illness or fever (temperature of 37.5°C or higher). All subjects enrolled spoke French as their primary language and interviews were conducted in French.

### 2.3. Demographics and Clinical Assessment

All participants provided demographic information, underwent a complete medical history, a general physical examination, and a thorough neurological assessment by neurologists at the Yaoundé Central Hospital to detect any focal neurological deficit suggestive of CNS opportunistic infection, before psychometric testing. This thorough clinical assessment of each subject combined with review of his or her prior medical history and subsequent laboratory data, ensured that potential confounding factors such as existing CNS opportunistic infections (OIs) were ruled out.

#### HIV serology, CD4 cell counts, and viral loads

Sample collection and all analyses were performed in the Hematology laboratory of the Yaoundé University Teaching Hospital, Cameroon. Venous blood samples were collected and stored at room temperature in the outpatient clinic and analyses performed in the Hematology laboratory within 6 hours of blood collection. The HIV status of each participant was determined using the rapid immunochromatographic HIV-1/2 test (Abbott Diagnostics, Chicago, IL, USA) and the Murex HIV antigen/antibody Combination ELISA (Abbott Diagnostics), according to the manufacturer’s instructions. A participant was considered HIV+ if he/she tested positive for the two tests, HIV- if negative for both tests, and discordant if positive for only one test. No discordant result was observed in this study.

Subjects’ CD4 T-lymphocyte counts were quantified by flow cytometry, using a Fluorescence Activated Cell Sorting (FACS) Count Instrumentation System and the BD FACSCount CD4 reagent kit (BD Biosciences, San Jose, CA, USA), according to the manufacturer’s instructions. The FACS instrument was calibrated and quality control tested before each experiment. For viral loads quantification, HIV RNA copy number in each plasma sample was quantified by reverse transcription polymerase chain reaction (RT-PCR), using Amplicor HIV-1 Monitor Test (Roche Diagnostic Systems, Pleasanton, CA), according to the manufacturer’s protocol. The assay detection limit was 40 viral copies / ml.

### 2.4. ART Adherence Questionnaire

The adult AIDS Clinical Trial Group (ACTG) adherence questionnaire [16] was translated into French and validated through back-translation [17]. This ART adherence questionnaire has been validated in population studies in both developed [18–20] and resources limited countries [20–23], and we previously adapted and validated this French version of the ACTG questionnaire in Cameroon [17]. This questionnaire was administered to each subject by a physician (JYF) in a face-to-face interview, and the same physician recorded all interview results. Participants were administered the ACTG questionnaire items that measure adherence behavior, including adherence to ART schedule and medication instructions over the course of their treatment. The physician-administered questionnaire form included the following questions: 1) have you ever missed ART? 2) Have you ever missed ART because of medication side effects? 3) Have you ever missed ART because of excessive drugs? 4) Have you ever had difficulties taking ART at the exact time? For each question, response was categorized into 4 levels of adherence, using a Likert scale: a) never, b) rarely, c) sometimes, and d) often. It was also recorded when patients had stopped taking ART medication, and “stopped” was considered as the 5^th^ level of adherence.

### 2.5. Adherence to ART Refills

The Yaoundé Central Hospital has a central pharmacy that dispenses ART medication to patients receiving medical care in the Day-care Service. Each refill is recorded in the hospital computer system, including subject ID, date, and time the prescription was collected by the patient. At each refill, the patient is given a month (30 days) ART supply and instructed to come back to the pharmacy for refill at day-25 (5 days before the drug supply provided runs out). We examined the hospital pharmacy records on September 28, 2015, to identify the individuals among the 171 subjects recruited in our study that also had pharmacy refills records. Our cut-off was any subject that had been on ART for a minimum of 6 months. The electronic pharmacy refill data was available for 50 of the 171 subjects (43 females and 7 males). Retrospective examination showed that all prescriptions and refills were made between February 4^th^, 2013 and September 7^th^, 2015, and these subjects had been on ART for 6.76 to 32.63 months (203 to 979 days). A subject was considered non-adherent if he/she had returned for refill over 2 days after the end of their 30-days ART supply (after day-32), thus had been non-adherent for a minimum of 3 days.

### 2.6. Assessment of Opportunistic Infections

We indirectly assessed the occurrence of OIs over the course of ART treatment by analyzing pharmacy refills and prescriptions for each of the 50 subjects. We therefore determined if and when antibiotics medication was prescribed and provided concurrently with ART refills or in between ART refills. We also determined the number of antibiotic refills for all 50 subjects during the 203 to 979 days ART treatment period, and the type of ART regimen before and during antibiotic prescriptions and antibiotic refills.

### 2.7. Beck Depression Inventory-II

The Beck Depression Inventory (BDI)-II [24, 25] is widely used to detect the presence of depressive moods and to measure the severity of depression. This instrument has been translated into many languages, including French, validated through back-translations and test-retest reliability. This validated French version of the BDI-II was shown to accurately diagnose depression in human studies in France [26, 27], Belgium [28], and Canada [29–31]. We previously adapted and validated this French version of the BDI-II in Cameroon [17]; and in our current study, this psychometric test was used to identify the presence and severity of depression. The BDI-II was administered to each subject by a trained psychometrist in a face-to-face interview. BDI-II consists of a 21-item questionnaire that assesses symptoms of depression; including affective symptoms such as hopelessness, irritability, feelings of guilt, pessimism, worthlessness, self-dislike, suicidal thought; and somatic symptoms such as sadness, crying, irritability, agitation, loss of interest, fatigue, difficulties sleeping and concentrating. The answer to each question is scored on a scaled value of 0 to 3, and the total score determines the severity of depression: 0–13 indicating minimal depression; 14–19 indicating mild depression; 20–28 indicating moderate depression; 29–63 indicating severe depression [24, 25].

### 2.8. Subjects Groups and Change in ART Regimen

Retrospective review of pharmacy refill records showed that for 50 of the 171 subjects who had pharmacy refill records, each had 4 to 31 ART refills, a combined total of 1160 ART refills, and the median number of days non-adherent during refills was 39 (range: 3 to 277 days). To analyze the effects of change in ART regimen on adherence, subjects were grouped into patients with a maximum of 3 to 39 days non-adherent at refills (GROUP1), and subjects with ≥ 40 days non-adherent at refills (GROUP2). To determine whether subjects had been non-adherent when their ART regimen was changed, we quantified the number of days they were non-adherent at or around the period their ART regimen was changed. For all subjects and all 1160 refills, the median number of days non-adherent at/or around the time of change in ART regimen was 28 (range: 0 to 272 days). For analysis, subjects were grouped into patients who were non-adherent for a maximum of 28 days at or around the time their ART regimen was changed (CHANGE1), and patients who were non-adherent for over 28 days at or around the time their ART regimen was changed (CHANGE2).

### 2.9. Statistical Analysis

The responses to the ACTG ART adherence questionnaire were compared between male and female patients using the Chi-square tests or Fisher’s exact tests. The responses to the adherence questionnaire and BDI-II scores were compared between the CD4 and viral load groups using the Chi-square tests, Fisher’s exact tests, Wilcoxon rank-sum tests, or Kruskal-Wallis Tests. For the pharmacy ART refill data, the binary outcome non-adherence status at each ART refill was assessed using logistic regressions for repeated measures of the Generalized Estimating Equations method. The length / duration of non-adherence at each refill was analyzed using negative binomial regressions for repeated measures of the Generalized Estimating Equations method. Dunnett-Hsu’s correction was used for multiple comparisons with a control group [32]. The binary outcomes non-adherence status was assessed using logistic regressions. A p-value <0.05 was considered statistically significant. All analyses were performed using SAS version 9.4 (SAS Institute, Inc., Cary, NC).

## Results

### 3.1. Demographics and Laboratory Characteristics

Cameroon has over 22 million inhabitants and the country’s 2015 HIV prevalence among adults aged 15 to 49 was 4.5% (2.9% among males, and 5.6% among females); females represented 60% of HIV-infected adults (15 to 49 years old), and 70% of HIV-infected youths (15 to 24 years old) [33, 34]. Of the 171 HIV-infected individuals recruited, 161 (131 females and 30 males) completed all the ACTG adherence questionnaires and the BDI-II. Their mean age was 39.9 ± 9 years; and 86 (53.42%) subjects were ≤ 40 years old, while 75 (46.58%) subjects were > 40 years old (Table 1). The mean number of years of education was 9.8 ± 3.8 years (Table 1). Fifty subjects (43 females and 7 males) had pharmacy ART records, as well as records of any other prescription from the Day-care Service. For those 50 subjects, the mean age was 42.6 ± 9.24 years, with 25 subjects ≤ 40 years old and 25 subjects > 40 years old; the mean number of years of education: 8.17 ± 3 years. CD4 cell counts were available for 137 subjects, and the mean CD4 cell counts was 458.7 ± 254.09 cells /μl, median 441 cells /μl; range 5 to 1657 cells /μl. Viral loads data was available for 139 subjects, including 32 subjects with detectable viral loads. The median log viral load was 4.31 (range: 1.64 to 6.14) log copies/ml.

**Table 1:** Subjects’ demographic and laboratory characteristics.

	Subjects (N = 161)
**Age (mean ± SD)**	39.9 ± 9
**Age > 40 years (N, %)**	75 (46.58)
**Age ≤ 40 years (N, %)**	86 (53.42)
**EDU (mean ± SD)**	9.8 ± 3.8
**EDU ≤ 10 years (N, %)**	99 (61.49)
**EDU 11 to 13 years (N, %)**	39 (24.22)
**EDU ≥ 14 years (N, %)**	23 (14.29)
**Females (N, %)**	131 (81.37)
**Males (N, %)**	30 (18.63)
**Mean CD4 ± SD (cells**/μl)	458 ± 254.09
**Speak French (N, %)**	161 (100)

EDU: education, SD: standard deviation; N: sample size.

EDU ≤ 10 years corresponds to a maximum middle school (8^th^ grade) level education.

EDU 11 to 13 years corresponds to a high school (9^th^ to 12^th^ grade) level education.

EDU ≥ 14 years correspond to college and post-graduate level education.

### 3.2. Analysis of Adherence Based on the ACTG Questionnaire

We analyzed a total of 171 cases on ART. Ten subjects had missing data and were not included in the analysis. Analysis of the remaining 161 subjects (131 females and 30 males) on ART using the Chi-squared test showed that ART side effects were associated with non-adherence (Table 2). Data further showed that males had a lower proportion of good adherence compared to females (76.67% vs. 90.84%), and a higher proportion of males (23.33%) than females (9.16%) admitted to having missed ART sometimes, or very often, or having stopped taking ART because of drugs’ side-effects (Table 2, p<0.05). There was no significant difference in the proportion of males and females that admitted to having been non-adherent due to excessive drugs or difficulties in taking their medication at the scheduled time (Table 2). Age and education did not influence ART adherence. There was no difference in the proportion of younger (≤40) subjects who were non-adherent, compared to the proportion of older (>40) subjects non-adherent. Similarly, there were no significant differences in the proportion of less educated subjects (maximum 12^th^ grade) who were non-adherent, compared to the proportion of more educated subjects (college and post-graduate) who were non-adherent.

**Table 2:** Self-reported adherence to ART based on ACTG questionnaire: gender effects.

Variable		Female (N = 131)	Male (N = 30)	P-value
Ever missed ART because of side effects?	Never or Rarely	119 (90.84)	23 (76.67)	0.05
	Sometimes, Often, or has Stopped	12 (9.16)	7 (23.33)	
Ever missed ART because of excessive drugs?	Never or Rarely	125 (95.42)	27 (90)	0.37
	Sometimes, Often, or has Stopped	6 (4.58)	3 (10)	
Ever had difficulty taking ART at the exact time?	Never or Rarely	109 (83.21)	25 (83.33)	0.99
	Sometimes, Often, or has Stopped	22 (16.79)	5 (16.67)	
Ever missed ART (for any reason)?	Never or Rarely	92 (70.23)	19 (63.33)	0.46
	Sometimes, Often, or has Stopped	39 (29.77)	11 (36.67)	

### 3.3. Having Low CD4 Cell Counts is Associated With Increased Risk of Non-Adherence

For subjects with available CD4 data, we performed additional analyses based on CD4 levels. Subjects with CD4 cell counts < 200 cells /μl had a lower proportion of good adherence, compared to subjects with CD4 cell counts ≥ 200 cells /μl (78.95% vs. 95.76%) (Table 3). A higher proportion of subjects with CD4 cell counts < 200 cells/ μl (21%) than subjects with CD4 cell counts ≥ 200 cells /μl (4.24%) admitted to having missed ART sometimes, or very often, or having stopped taking ART because of excessive drugs (Table 3, p = 0.02). Additional analyses confirmed these findings and showed that subjects with CD4 cell counts < 350 cells/ μl had a lower proportion of good adherence, compared to subjects with CD4 cell counts ≥ 350 cells/ μl (78 to 85% vs. 90 to 96.88%) (Table 4). A higher proportion of subjects with CD4 cell counts < 350 cells /μl (21.95%) than subjects with CD4 cell counts ≥ 350 cells /μl (9.38%) admitted to having missed ART sometimes, or very often, or having stopped taking ART because of side effects (Table 4, p = 0.046). Similarly, A higher proportion of subjects with CD4 cell counts < 350 cells /μl (14.63%) than subjects with CD4 cell counts ≥ 350 cells /μl (3.13%) admitted to having missed ART sometimes, or very often, or having stopped taking ART because of excessive number of drugs (Table 4, p = 0.02).

**Table 3:** Self-reported adherence to ART based on ACTG questionnaire: effects of CD4 cell levels [CD4 <200 cells /μl (group 1) vs. CD4 ≥ 200 cells /μl (groups 2, 3, and 4)].

Variable		CD4 group1 (N = 19)	CD4 groups 2+3+4 (N = 118)	P-value
Ever missed ART because of side effects?	Never or Rarely	14 (73.68)	105 (88.98)	0.13
	Sometimes, Often, or has Stopped	5 (26.32)	13 (11.02)	
Ever missed ART because of excessive drugs?	Never or Rarely	15 (78.95)	113 (95.76)	**0.02**
	Sometimes, Often, or has Stopped	4 (21.05)	5 (4.24)	
Ever had difficulty taking ART at the exact time?	Never or Rarely	15 (78.95)	97 (82.2)	0.75
	Sometimes, Often, or has Stopped	4 (21.05)	21 (17.8)	
Ever missed ART (for any reason)?	Never or Rarely	11 (57.89)	80 (67.8)	0.4
	Sometimes, Often, or has Stopped	8 (42.11)	38 (32.2)	

**Table 4:** Self-reported adherence to ART based on ACTG questionnaire: effects of CD4 cell levels [CD4 <350 cells /μl (groups 1 and 2) vs. CD4 ≥ 350 cells /μl (groups 3 and 4)].

Variable		CD4 groups1+2 (N = 41)	CD4 groups3+4 (N = 118)	P-value
Ever missed ART because of side effects?	Never or Rarely	32 (78.05)	87 (90.63)	**0.046**
	Sometimes, Often, or has Stopped	9 (21.95)	9 (9.38)	
Ever missed ART because of excessive drugs?	Never or Rarely	35 (85.37)	93 (96.88)	**0.02**
	Sometimes, Often, or has Stopped	6 (14.63)	3 (3.13)	
Ever had difficulty taking ART at the exact time?	Never or Rarely	33 (80.49)	79 (82.29)	0.8
	Sometimes, Often, or has Stopped	8 (19.51)	17 (17.71)	
Ever missed ART (for any reason)?	Never or Rarely	26 (63.41)	65 (67.71)	0.63
	Sometimes, Often, or has Stopped	15 (36.59)	31 (32.29)	

Subject classification based on CD4 counts:

Group 1: Severe (severe immunosuppression): CD4 ≤ 200 cells /μl.

Group 2: Advanced (advanced immunosuppression): CD4 between 200 and 349 cells /μl.

Group 3: Mild (mild immunosuppression): CD4 between 350 and 499 cells /μl.

Group 4: No immunosuppression: CD4 ≥ 500 cells /μl.

### 3.4. Having High Viral Loads is Associated With Increased Risk of Non-Adherence

For subjects with available viral loads data, we performed additional analyses based on the levels of viremia. Subjects with detectable viral loads had a lower proportion of good adherence compared to subjects with undetectable viral loads (71.88% vs. 91.59%) (Table 5). A higher proportion of subjects with detectable viral loads (28.13%) than subjects with undetectable viral loads (8.41%) admitted to having missed ART sometimes, or very often, or having stopped taking ART because of side effects (Table 5, p = 0.007).

**Table 5:** Self-reported adherence to ART based on ACTG questionnaire: effects of viral loads.

Variable		VL groups1+2 (N = 32)	VL group3 (N = 107)	P-value
Ever missed ART because of side effects?	Never or Rarely	23(71.88)	98(91.59)	**0.007**
	Sometimes, Often, or has Stopped	9(28.13)	9(8.41)	
Ever missed ART because of excessive drugs?	Never or Rarely	28(87.5)	102(95.33)	0.21
	Sometimes, Often, or has Stopped	4(12.5)	5(4.67)	
Ever had difficulty taking ART at the exact time?	Never or Rarely	23(71.88)	91(85.05)	0.09
	Sometimes, Often, or has Stopped	9(28.13)	16(14.95)	
Ever missed ART (for any reason)?	Never or Rarely	18(56.25)	75(70.09)	0.14
	Sometimes, Often, or has Stopped	14(43.75)	32(29.91)	

Subject classification based on viral load (VL):

Group 1: High; viral load ≥ 100,000 copies /ml.

Group 2: Mid-Range; viral load >40 and <100,000 copies /ml.

Group 3: Non-detectable; viral load <40 viral copies /ml.

### 3.5. Analysis of ART Adherence Based on Pharmacy Refills Data

Data on pharmacy ART refills was available for 50 subjects (6.7 males and 43 females) who had been on ART for 6.7 to 32.6 months: 8 subjects had been on ART for 7 to 20 months, and 42 subjects had been on ART for 23 to 32.6 months. The number of ART refills for the 50 subjects varied from 4 to 31. This included 5 subjects with 4 to 8 refills, 8 subjects with 11 to 20 refills, and 37 subjects with 21 to 31 refills. The number of non-adherent refills per subject varied from 1 to 15. Only 4 subjects had 1 to 2 non-adherent refills during that treatment period and 8 to 66.6% of refills were non-adherent. For the 50 subjects, the total number of days on ART varied from 203 to 979 days, the number of days non-adherent varied from 3 to 381 days (0.9 to 56%). The 50 subjects had a total of 1210 ART prescriptions and 1160 ART refills and 406 refills (35%) were non-adherent. ART consisted of a total of 9 different regimens (Table 6), but only 3 regimens were regularly prescribed; 97.35% of all ART prescriptions and refills consisted of regimens R1 (AZT/3TC/NVP), R2 (TDF/3TC/NVP), or R3 (TDF/3TC/EFV) (Table 6).

**Table 6:** Patients’ ARV drug regimens.

Regimen	ARV drugs	Number of Subjects	Prescriptions and refills (N, %)
R1	AZT/3TC/NVP	35	427 (35.29%)
R2	TDF/3TC+NVP	9	151 (12.48%)
R3	TDF/3TC+EFV	41	600 (49.58%)
R4	AZT/3TC/EFV	5	11 (0.9%)
R5	TDF/3TC+ATV/r	1	4 (0.33%)
R6	TDF/3TC+/LPV/r	1	5 (0.4%)
R7	TDF/3TC+ABC+ LPV/r	1	1 (0.08%)
R8	AZT/3TC+/LPV/r	1	10 (0.82%)
R9	AZT/3TC	1	1 (0.08%)

Abbreviations: N: sample size; ARV: antiretroviral; AZT: zidovudine; 3TC: lamivudine; NVP: Nevirapine; TDF: tenofovir; EFV: efavirenz; ATV/r: atazanavir/ritonavir; LPV/r, lopinavir/ritonavir; ABC: abacavir.

### 3.6. Having Opportunistic Infections is Associated With Increased Non-Adherence to ART

To estimate the proportion of patients that may have had OIs during the 6.7 to 32.6 months ART treatment period, we analyzed all prescriptions to identify subjects who had been prescribed or given antibiotics at ART refills or in between refills. Nineteen (38%) of the subjects had been prescribed antibiotics over the course of their ART treatment, mostly cotrimoxazole, with the number of antibiotic prescriptions varying from 1 to 5. Compared to patients on ART without OIs, patients with OIs (antibiotics treatment) had 2.42 times higher odds of having been non-adherent, as shown by univariate logistic regression analysis (Table 7: odds ratio (OR) 2.42, 95% confidence interval (CI): 1.43 to 4.12, p<0.001). Multivariable logistic regression analysis adjusting for the type of ART regimen, education, gender, age, and depression, confirmed these findings and showed that patients who had been on antibiotics treatment had 3.1 times higher odds of having been non-adherent, compared to patients who had not taken antibiotics (Table 8, 95% CI: 1.69 to 5.68, p = 0.0003).

**Table 7:** Effect of ART regimens, opportunistic infections, and age on non-adherence to ART: univariate logistic regression analysis.

Variables		Odds ratios	95% Confidence Interval		P-value
ART regimens	R1	0.85	0.65	1.10	0.29
	R2	0.85	0.56	1.29	0.60
	R3	Reference			
ART regimens	R1	0.999	0.68	1.46	0.99
	R2	Reference			
Antibiotics	Yes	2.42	1.43	4.12	**0.001**
	No	Reference			

**Table 8:** Effect of ART regimens, opportunistic infections, and age on non-adherence to ART: multivariable logistic regression analysis.

Variables		Odds ratios	95% Confidence Interval		P-value
ART regimens	R1	0.79	0.59	1.06	0.15
	R2	0.89	0.63	1.27	0.70
	R3	Reference			
EDUC years		0.99	0.93	1.05	0.67
Gender	F	1.44	0.66	3.13	0.36
	M	Reference			
Antibiotics	Yes	3.10	1.69	5.68	**0.0003**
	No	Reference			
Age	>40	0.66	0.47	0.92	**0.01**
	≤40	Reference			
Beck total		1.002	0.99	1.02	0.78

ART: Antiretroviral therapy; R: ART regimen, EDUC: education; F: females; M: males

To determine whether these results could be influenced by longer treatment duration (subjects who had higher number of refills), we performed additional analyses including only data from the first 11 refills (6.7 to 12 months’ treatment) for each subject. Univariate logistic regression analysis of data from the first 11 refills showed that patients with OIs had 3.26 times higher odds of having been non-adherent (95% CI: 1.67 to 6.36, p = 0.0006). Multivariable logistic regression analysis of these data, adjusting for the type of ART regimen, education level, gender, age, and depression, showed that patients with OIs had 4.82 times higher odds of having been non-adherent (95% CI: 2.18 to 10.65, p = 0.0001).

### 3.7. Having Opportunistic Infections is Associated With Increased Duration of the Non-Adherence Period

Univariate regression analysis showed that patients who had received antibiotic treatment had higher number of non-adherent days (Table 9), and multivariable analysis adjusting for the type of ART regimen, education, gender, age, and depression, confirmed these results and showed that antibiotics treatment was significantly associated with higher numbers of non-adherent days (p = 0.02, Table 10). To determine whether these results could be influenced by longer treatment duration, we performed additional analyses including only data from the first 11 refills for each subject. Antibiotics treatment was significantly associated with higher numbers of non-adherent days by univariate regression analysis (p = 0.03, 95%CI: 0.09 to 1.81), as well as by multivariable regression analysis adjusting for the type of ART regimen, education level, gender, age, and depression (p = 0.001, 95%CI: 0.48 to 1.97).

**Table 9:** Effect of ART regimens and opportunistic infections on the length of non-adherence (number of non-adherent days): univariate negative binomial regression analysis.

Variables		Coefficient*	95% Confidence Interval		P-value
ART regimens	R1	-0.17	-0.66	0.31	0.66
	R2	-0.56	-1.04	-0.07	**0.02**
	R3	Reference			
ART regimens	R1	0.38	-0.19	0.96	0.24
	R2	Reference			
Antibiotics	Yes	0.62	-0.08	1.31	**0.08**
	No	Reference			

**Table 10:** Effect of ART regimens and opportunistic infections on the length of non-adherence (number of non-adherent days): multivariable negative binomial regression analysis.

Variables		Coefficient*	95% Confidence Interval		P-value
ART regimens	R1	-0.13	-0.56	0.31	0.74
	R2	-0.33	-0.73	0.06	0.11
	R3	Reference			
EDUC years		-0.02	-0.08	0.03	0.41
Gender	F	0.11	-0.45	0.66	0.70
	M	Reference			
Antibiotics	Yes	0.79	0.15	1.43	**0.02**
	No	Reference			
Age	>40	-0.74	-1.10	-0.39	**<0.0001**
	≤40	Reference			
Beck total		0.02	-0.04	-0.0005	**0.04**

ART: Antiretroviral therapy; R: ART regimen, EDUC: education; F: females; M: males

### 3.8. Effects of ART Regimen on Adherence

Pharmacy prescriptions and refills data were available for 50 subjects, who had received a total 1210 ART prescriptions and 1160 ART refills over 6.7 to 32.6 months. Only 2 subjects had received 2^nd^ line ART (a combined total of 20 prescriptions and refills of 2^nd^ line ART); 1190 (98.34%) of ART prescriptions were 1^st^ line ART, and 97.35% of regimens consisted of R1 (AZT/3TC/NVP), R2 (TDF/3TC/NVP), or R3 (TDF/3TC/EFC) (Table 6). Therefore, we performed analyses to determine whether any of these 3 regimens was associated with non-adherence. Both univariate and multivariable logistic regression analyses showed that the type of ART regimen was not associated with non-adherence (Tables 7 and 8). Univariate negative binomial regression for repeated measures was performed to determine the effects of ART regimen on the length of non-adherence. Data showed that compared to subjects on R3 regimen, subjects on R2 regimen had lower log number of non-adherent days (Table 9, p = 0.02). This association was not significant when only data for the first 11 refills were considered (data not shown). There was no significant difference in the length of non-adherence between subjects on R1 and those on R3 regimens, or between subjects on R1 and those on R2 regimens (Table 9). Multivariable regression analysis adjusting for education level, gender, age, antibiotics treatment, and depression, showed no significant effect of ART regimen on the length of non-adherence (Table 10).

### 3.9. Increased Risk of Non-Adherence to ART Among Younger HIV-Infected Cameroonians

Multivariable logistic regression analysis after adjusting for ART regimen, education level, gender, antibiotics use, and depression, showed that compared to younger subjects (≤ 40 years), older subjects (>40 years) were significantly less likely to be non-adherent (OR: 0.66, 95% CI: 0.47 to 0.92; p = 0.01) (Table 8). Additional multivariable analyses adjusting for ART regimen, education level, gender, antibiotics use, and depression, showed that compared to younger subjects, older subjects had significantly fewer numbers of non-adherent days (p<0.0001, Table 10).

To determine whether subjects with longer treatment duration could influence these results, we performed additional analyses including only data from the first 11 refills (6.7 to 12 months’ treatment) for each subject. This second analysis also showed that compared to younger subjects, older subjects had significantly fewer numbers of non-adherent days (p<0.05, 95%CI: -1.25 to -0.005).

### 3.10. Association of Depression Symptoms With Non-Adherence to ART, the Length of the Non-Adherence Period, and CD4 Cell Counts

Univariate analysis of Beck total scores using Wilcoxon rank-sum test showed higher median Beck total scores among females compared to males (p = 0.04), and among subjects with less than 14 years (maximum 12^th^ grade) of education, compared to subjects with ≥14 years (college and post-graduate) of education (p = 0.052). Higher number of days non-adherent at the time of antibiotic refills was associated with higher Beck total scores, and Spearman correlation analysis showed a significant positive correlation between Beck total scores and the number of days non-adherent at the time of antibiotic refill (r = 0.53, p = 0.04). Further univariate analyses of Beck total scores using Wilcoxon rank-sum test showed higher median Beck total scores among subjects with CD4 cell counts < 350 cells/ μl, compared to subjects with CD4 cell counts ≥ 350 cells/ μl (Table 11, p = 0.04). Multivariable logistic regression analysis adjusting for the type of ART regimen, education level, antibiotics treatment, gender, and age showed that the degree of depression symptoms (higher Beck total scores) was significantly associated with increased number of days non-adherent (p = 0.04, Table 10).

**Table 11:** Effect of CD4 count and viral loads on the risk of depression (BDI-II).

Outcome	Variables		N	Median Score	Range	P-value
**BECK Total Score**	CD4 (cells /μl)	CD4 < 350	39	18	0–51	0.04
		CD4 ≥ 350	95	13	0–46	
	Viral load (copies/ml)	VL ≥ 40 (Detectable)	30	13	0–50	0.59
		VL < 40 (Undetectable)	106	14	0–51	

### 3.11. Change in ART Regimen is Associated With Increased Likelihood of Non-Adherence

For all subjects and all 1160 ART refills, the maximum number of days non-adherent during refills varied from 3 to 277 days (median = 39 days). The number of days non-adherent at or around the time of change in ART regimen varied from 0 to 272 days (median = 28 days). Logistic regression analyses showed an association between the duration of non-adherence to refill and the number of days subjects were non-adherent at the time of change in ART regimen. Subjects who had fewer than 29 days of non-adherence at the time of change in ART regimen (CHANGE1) were more likely to have a lower number of non-adherent days (3 to 39) to refill (GROUP1), and subjects with over 28 (29 to 272) days of non-adherence at the time of change in ART regimen (CHANGE2) were 4.59 times more likely to have higher number of non-adherent days (40 to 277 days) to refill (GROUP2) (95% CI: 1.39 to 15.15, p = 0.01) (Table 12).

**Table 12:** Higher numbers of days non-adherent during change in ART regimen correlate with increased non-adherence and longer time of non-adherence at a refill. (Logistic regression analysis).

Outcome	Effect	Odds Ratio	95% Confidence Interval		P-value
Non-adherent days group	ART change groups				
	CHANGE2	4.59	1.39	15.15	0.01
	CHANGE1	Reference			

Multiple logistic regression analyses indicated that there was no evidence of an interaction between age and the length of non-adherence at the time of change in ART regimen (CHANGE1 or CHANGE2), and showed that after adjusting for age, subjects with over 28 days of non-adherence at the time of change in ART regimen were 5.33 times more likely to have over 39 days of non-adherence to refill (95% CI: 1.51 to 18.79, p = 0.009) (Table 13).

**Table 13:** Higher numbers of days non-adherent during change in ART regimen correlate with increased non-adherence and longer time of non-adherence at a refill. (Logistic regression analysis controlling for age).

Outcome	Effect	Odds Ratio	95% Confidence Interval		P-value
Non-adherent days group	ART change groups				
	CHANGE2	5.33	1.51	18.79	0.009
	CHANGE1	Reference			

## Discussion

According to WHO and UNAIDS “Treatment 2015” program, Cameroon is one of the 20 SSA countries where 9 of every 10 HIV-infected people have unmet ART needs [8]. There has been a gradual scale-up of HIV treatment in Cameroon and ART coverage among Cameroonians living with HIV/AIDS increased from 0% in 2003 to 22% in 2014 [5, 6]. However, good adherence to ART is necessary to prevent virologic failure, diminish the risk of drug resistance and viral transmission to uninfected sexual partners, and prevent disease progression and HIV/AIDS-related deaths. Overall, good adherence is required for the success of HIV treatment programs; poor adherence is associated with virologic failure and it is estimated that > 90% adherence is necessary to adequately suppress viral replication and stop disease progression [35].

There is evidence of poor adherence, treatment interruptions, and loss to follow-up among HIV+ subjects on ART in Cameroon [9–14]. Identifying the barriers to adherence in Cameroon is necessary for the development and implementation of effective ART adherence strategies. Using both patients’ self-reported questionnaires and pharmacy refill data, we show here that non-adherence to ART in Cameroon is associated with the presence of drug side-effects, OIs, lower CD4 cell counts, higher viral loads, and depression symptoms, and are associated with change in ART regimen but not with the type of ART regimen.

The methods used to assess adherence in our studies were previously validated and shown to reliably measure ART adherence in other studies [7, 21–23]. In fact, it has been shown that regular attendance to pharmacy refills and clinic visit appointments is associated with retention in care and better adherence to ART [36], predicts viral suppression, and that sub-optimal attendance predicts virologic failure [9, 10, 21, 23, 37] and drug resistance [15]. In our study, a subject was considered non-adherent for a refill if he/she had missed pharmacy refill for over 48 hours. This cut-off has been validated in previous studies [7, 38]. A study of 4489 adults on ART in three SSA countries (Tanzania, Uganda, and Zambia) showed that missing ART for 48 hours was strongly associated with failure of viral control [38].

Females have been reported to represent 60% of HIV-infected adults, and 70% of HIV-infected youths in Cameroon [33, 34], but in our current study on ART adherence, 81% of subjects were females. This is consistent with the gender difference observed in other ART programs across several countries in SSA. Retrospective and prospective cohort studies in several SSA countries showed that despite the availability of free ART, significantly more females (compared to males) were enrolled in ART treatment programs, males enrolled were at more advanced AIDS stage, and loss to follow-up was significantly higher in males [39, 40]. This gender difference in HIV care affects therapeutic outcomes, as HIV-infected African men are significantly more likely to experience virologic failure and higher mortality [41–43].

A significant number of subjects in our study admitted missing ART because of drug side effects. Data from cohort studies in other countries also showed that ART-related side effects is a strong predictor of non-adherence [16, 44–47], low retention in care [48] and discontinuation of ART [44]. Several population-based cohort studies in Western countries [49, 50], and other SSA countries [51, 52] showed poor adherence to ART among males and younger subjects, compared to females and older subjects, and this was associated with lower probability of retention in care [50], increased risk of ART discontinuation [49] and loss to follow-up [53]. Our current study also shows gender and age effects on adherence to ART in Cameroon. Compared to females, males were significantly more likely to forego ART due to side effects or fear of side effects; and compared to younger individuals (≤40), older subjects were less likely to be non-adherent and had significantly shorter duration of non-adherence. This suggests that targeted interventions focusing specially on males and younger HIV-infected subjects in Cameroon may improve adherence to ART. Such targeted interventions could include counseling and reminder tools; a study of 232,389 HIV-infected subjects initiating ART at 349 different clinics in ten SSA countries showed that providing counseling and educational materials to subjects resulted in lower attrition and fewer subjects lost to follow-up [54].

The progression of HIV infection and advance to AIDS is characterized by the occurrence of OIs, including bacterial infections requiring antibiotic treatment. For patients with available pharmacy refill data, we assessed the occurrence of OIs by analyzing antibiotics prescriptions and refills. Thirty eight percent of the subjects had received antibiotic prescriptions and refills over the course of their ART treatment. The antibiotic most commonly prescribed was cotrimoxazole, a combination of trimethoprim and sulphamethoxazole that is used for the treatment of several bacterial, parasitic, and fungal infections, including pneumocystis pneumonia, toxoplasmosis, shigellosis and bronchitis [55, 56]. This antibiotic is recommended by the WHO as the treatment of choice against OIs for HIV-infected individuals [7]. We demonstrated that having antibiotic prescription was significantly associated with increased risk of non-adherence to ART and longer non-adherent periods, suggesting that non-adherence may have been due to OIs and other HIV/AIDS-related complications. In fact, maximal periods of non-adherence were often observed at /or around the time of antibiotic prescription. It is also possible that increased non-adherence could lead to disease progression and increased susceptibility to OIs, necessitating antibiotic treatment, and treatment failure, necessitating changes of ART regimens. Consistent with that, in our current study, change in a patient’s ART regimen often occurs around the time of maximal length of non-adherence, and antibiotic prescription. Using logistic regression analysis, we showed that having longer or extended periods of non-adherence was associated with an increased likelihood of change in ART regimens at the time of antibiotic prescription, and this association remained statistically significant after controlling for age. This suggests that the extended periods of non-adherence, were not only associated to OIs, but may also be associated to increased risks of failure of ongoing ART regimens. The fact that analysis of data from only the first 11 refills showed significant increase in the risk and duration of non-adherence in subjects with OIs confirmed the association of OIs and non-adherence to ART, and showed that our data was not biased by subjects with longer treatment duration. Although drinking alcohol and smoking are known risk factors for non-adherence to ART, they likely did not play a role in our results because current alcohol intoxication was an exclusion criterion, and only 3.8% of our subjects tested positive for nicotine.

Depression has been associated with non-adherence to ART. A review of 111 studies including 42,366 HIV/AIDS subjects from both developed and resource-limited countries [57], as well as a 2^nd^ review of 207 studies [58], showed that higher prevalence of depression symptoms was associated with lower likelihood of adherence to ART. Our current study showed higher levels of depression symptoms among females, compared to males, and there was no interaction between gender and Beck depression scores. Our current data also showed a strong positive correlation between the presence of depression symptoms and extended periods of non-adherence due to OIs (increased length of non-adherence at antibiotic refills). We also showed that the presence of depression symptoms was associated with lower CD4 cell counts and an overall increase in the length of non-adherence. In addition to their association with non-adherence to ART, depressed moods among HIV-infected Cameroonians can also contribute to virologic failure, as studies of HIV-infected subjects in other countries showed that subjects with poor adherence and those feeling depressed are more likely to have unsuppressed viral loads [59, 60]. A recent pilot study of 41 depressed HIV patients in Bamenda, Cameroon also showed that care and treatment of depression could improve HIV clinical outcomes and reduce the number of missed ART doses [61], further indicating the potential virologic and behavioral benefits of depression care among HIV-infected Cameroonians.

## Conclusions and Recommendations

In summary, our data confirmed previous findings [9–14] of poor adherence to ART in Cameroon. We showed that this non-adherence is associated with the presence of depressed moods. This suggests that strategies aimed at providing care for mental health issues such as depression to subjects enrolled in Cameroon ART programs could improve adherence. We also demonstrated that non-adherence was associated with the presence of ART drug side effects, and may result from these side effects. As patients may not report side effects if they are not asked, clinicians at HIV treatment centers could adopt a policy to systematically asking HIV-infected subjects during their visits whether they had experienced any side effects, and closely monitor all subjects with side effects. Counseling and taking appropriate measures to decrease these adverse events may increase adherence to ART and HIV treatment outcomes.

Our data showed a strong association between antibiotics treatment and non-adherence to ART, suggesting that non-adherence to ART in Cameroon is associated with increased incidence of OIs. Clinicians at HIV treatment centers could adopt a policy to systematically follow-up subjects on antibiotics or with OIs, to ensure timely and adequate treatment of these OIs, while simultaneously using other reminder tools to improve adherence to ART. The fact that males were more likely to forego ART because of side effects, that younger subjects were significantly more likely to be non-adherent and for a longer duration, suggests that specific attention should be paid to males and younger subjects in these targeted interventions to improve adherence to ART.

## Study Limitations

The limitations of this study included its small sample size and uneven number of male and females. We could only access antibiotic medications from the Yaoundé Central hospital pharmacy records, and do not know whether patients could have also obtained antibiotics from a different pharmacy or other / different health care providers. We cannot determine the sequence of events: we do not know whether OIs lead to non-adherence, or whether non-adherence leads to virologic failure and the development of OIs and AIDS-related symptoms. Similarly, we cannot establish with certainty that depressed moods lead to non-adherence, or non-adherence and OIs cause further stress and depression. It is also not known what proportion of delayed pharmacy ART refills in our study may have been due to ART stock-outs, compared to subjects being just non-adherent to pharmacy refill appointments. It is possible that medicine stock-outs could have played a role, as a recent survey of 15 ART treatment centers in Cameroon showed that only 14% had reached the desirable WHO levels of no pharmacy stock-outs [62]. However, we did not observe a pattern of simultaneous non-adherent refills by several subjects during the same period of time, suggesting that pharmacy stock-outs were not the principal cause of non-adherence for these subjects. Nevertheless, ensuring constant supply and availability of ART drugs can only improve adherence and ensure the success of HIV treatment programs.
